# High Levels of KAP1 Expression Are Associated with Aggressive Clinical Features in Ovarian Cancer

**DOI:** 10.3390/ijms16010363

**Published:** 2014-12-26

**Authors:** Yanfen Cui, Shaobin Yang, Xin Fu, Jingwen Feng, Shilei Xu, Guoguang Ying

**Affiliations:** 1 Laboratory of Cancer Cell Biology, National Clinical Research Center for Cancer, Tianjin Key Laboratory of Cancer Prevention and Therapy, Tianjin Medical University Cancer Institute and Hospital, Tianjin 300060, China; E-Mails: cuiyanfen@163.com (Y.C.); hbyangshaobin@163.com (S.Y.); j_w_feng@163.com (J.F.); slxu@sibs.ac.cn (S.X.); 2 Department of Gynecology Cancer, National Clinical Research Center for Cancer, Tianjin Key Laboratory of Cancer Prevention and Therapy, Tianjin Medical University Cancer Institute and Hospital, Tianjin 300060, China; E-Mail: fxhappy@126.com

**Keywords:** KAP1, ovarian cancer, prognostic factor

## Abstract

KAP1 is an universal corepressor for Kruppel-associated box zinc finger proteins in both normal and tumor cells. In this study, the biological function and clinical significance of KAP1 expression in ovarian cancer were investigated. Immunohistological staining of KAP1 was evaluated in 111 patients with ovarian epithelial cancer, 15 with ovarian borderline tumor, and 20 normal ovarian tissue. The correlations of KAP1 expression with clinicopathological features were studied. Kaplan-Meier analysis and Cox proportional hazard modeling were used to assess overall survival to analyze the effect of KAP1 expression on the prognosis of ovarian cancer. The positive rates of KAP1 were significantly higher in ovarian epithelial cancer (55.7%) and borderline tumor (20.0%) than in normal ovarian tissue (5.0%) (all *p* < 0.01). KAP1 expression correlated significantly with clinical stage (χ^2^ = 14.57, *p* < 0.0001), pathological grade (χ^2^ = 6.06, *p* = 0.048) and metastases (χ^2^ =10.38, *p* = 0.001). Patients with high KAP 1 levels showed poor survival (*p* < 0.0001). Multivariate analysis showed that KAP1 high expression was an independent predictor for ovarian cancer patients (hazard ratio = 0.463; 95% confidence interval = 0.230–0.9318, *p* = 0.031). Functionally, depletion of KAP1 by siRNA inhibited ovarian cancer cell proliferation, cell migration. KAP1 expression correlated with aggressive clinical features in ovarian cancer. High KAP1 expression was a prognostic factor of ovarian cancer.

## 1. Introduction

Epithelial ovarian cancer is the deadliest gynecological malignancy and the second leading cause of cancer-related deaths among women worldwide [1]. About 22,240 women were diagnosed with invasive epithelial ovarian cancer in the United States in 2013 [2]. Ovarian cancer is relatively uncommon in China, but an increase in the incidence has been reported. Statistics from the Tianjin Medical University Cancer Institute and Hospital showed that the incidence of ovarian cancer was 9.71/100,000 during the period of 1999–2010 in China [3]. Since ovarian cancer has no early typical symptoms and effective diagnostic methods, and is located deep within the pelvis and difficult to assess, more than 70% of patients are diagnosed at an advanced stage [4]. As a result, the prognosis of ovarian cancer patients is very poor. Therefore, it is urgent to find new biomarkers, which are able to identify specific patients who may benefit from aggressive therapies and predict the prognosis of epithelial ovarian cancer [5,6].

KRAB-associated protein 1 (KAP1, also known as TRIM28 and TIF1b), is a well-known transcriptional corepressor of Kruppel-associated box zinc finger protein [7]. KAP1 is an essential partner in several multiple-protein complexes and involves a wide range of biological processes. It is critical for epigenetic stability during mouse oocyte to embryo transition [8] and convergent extension of extra-embryonic tissues [9]. KAP1 is implicated in repression of endogenous retroviruses in mouse embryonic stem cells [10]. Additionally, ATM-mediated phosphorylation of KAP1 leads to its co-localization with several proteins controlling DNA damage repair in response to genotoxic stress [11,12]. Phosphorylated KAP1 functions in derepression of its target genes [13,14]. Moreover, the CBF-A/KAP1/FTS-1 complex activates FSP-1 transcription and subsequent epithelial-mesenchymal transition [15].

Although a growing number of studies have demonstrated the function of KAP1, no reports have shown the expression status of KAP1 in ovarian cancer and any clinical significance associated with KAP1 expression. We report here that KAP1 is highly expressed in ovarian cancer tissues and is a potential novel prognosis marker for ovarian cancer patients.

## 2. Results

### 2.1. Higher Expression of KAP1 Protein Detected in Human Ovarian Cancer Tissues

**Figure 1:**
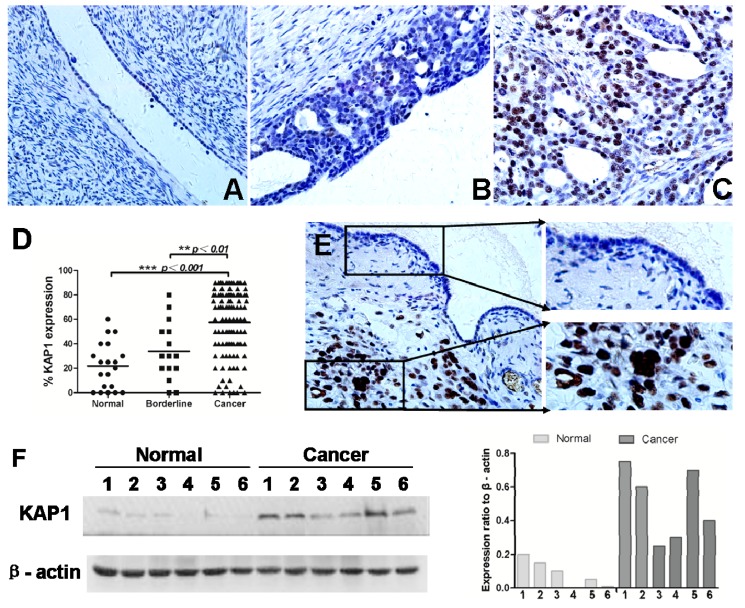
Expression of KAP1 in human non-tumor ovarian tissues and ovarian cancer. (**A**) in normal ovarian tissue; (**B**) in borderline tumor; (**C**) in ovarian cancer; (**D**) KAP1 expression levels in normal ovarian, borderline tumor and ovarian cancer tissues; (**E**) the expression level of KAP1 in non-tumor cells was lower than tumor cells in one sample and (**F**) western blot showed the expression level of KAP1 in cancer tissues was higher than normal ovarian tissues. (IHC, 400×). (** *p* < 0.01; *** *p* < 0.001).

To investigate the expression of KAP1 in ovarian cancer tissues and normal ovarian tissues, immunohistochemistry (IHC) was applied to show that KAP1 was expressed in both tumor cells and mesenchymal cells, and localized to the cell nucleus (Figure 1). Furthermore, we compared the expression level of KAP1 in 111 patients with ovarian epithelial cancer, 15 with ovarian borderline tumor, and 20 normal ovarian tissue. The results showed that the expression level of KAP1 was higher in ovarian cancer samples than non-tumor ovarian tissues (Figure 1A–D), and the positive rates of KAP1 were significantly higher in ovarian epithelial cancer (55.7%) and borderline tumor (20.0%) than in normal ovarian tissue (5.0%) (all *p* < 0.01) (Table 1). It also showed that the level of KAP1 in cancer tissues was higher than adjacent normal tissues in the same ovarian cancer sample (Figure 1E).

In addition, we selected six freshly frozen ovarian cancer tissues and six normal tissues randomly to detect the expression level of KAP1 using western blot analysis. It was found that the expression level of KAP1 in cancer tissues was higher than in normal tissues (Figure 1F).

**Table 1:** Expression of KAP1 in different ovarian tissues.

Groups	Cases	KAP1 Expression		High Rates (%)	*t*	*p*
		Low	High			
Normal	20	19	1	5.0	5.57	<0.001 *
Borderline	15	12	3	20.0	1.68	0.10 ***
Cancer	111	47	64	57.7	3.14	0.002 **

* Compared with the cancer group; ** Compared with the borderline group; *** Compared with the Normal group.

### 2.2. KAP1 Expression Levels in Different Histological Types of Ovarian Cancer

**Figure 2:**
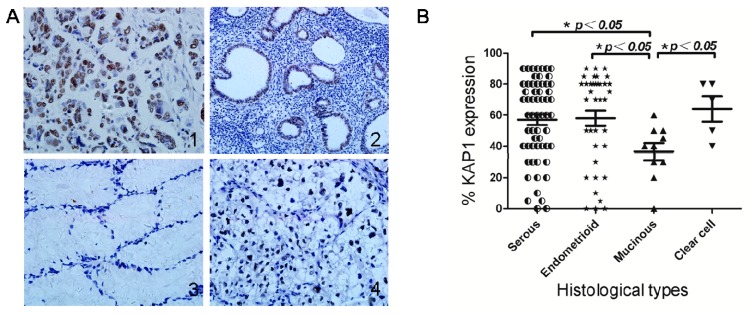
KAP1 expression levels in different histological types (**A**1, serous carcinomas, **A**2 endometrioid carcinomas, **A**3 mucinous carcinomas and **A**4 clear cell adenocarcinomas); (**B**) Significant differences in KAP1 expression levels between serous, endometrioid carcinoma or clear cell adenocarcinoma and mucinous carcinoma. (* *p* < 0.05).

In this study, we gathered 111 ovarian cancer cases, including 59 serous carcinomas, 37 endometrioid carcinomas, nine mucinous carcinomas and six clear cell adenocarcinomas. We examined KAP1 expression levels in different histological types (Figure 2). The average percentage of KAP1-positive cancer cells was 57.03% (range = 0%–94.0%) in serous carcinomas, 57.97% (range = 0%–90%) in endometrioid adenocarcinomas, 61.67% (range = 40.0%–82.0%) in clear cell adenocarcinomas, and 35% (range = 0%–60.0%) in mucinous carcinomas. The KAP1 expression level was significantly lower in mucinous carcinomas than other ovarian cancer types (all *p* < 0.05).

### 2.3. KAP1 Expression and Its Correlation with Clinicopathologic Variables

We also analyzed the association of KAP1 expression levels with clinicopathological variables of cancer, including age, Federation of Gynecology and Obstetrics (FIGO) stage, histology grade, ascites production and metastasis status. The results were summarized in Table 2. The expression levels of KAP1 protein measured by means of IHC were significantly associated with a higher clinical stage (χ^2^ = 14.57, *p* = 0.000), higher histological grade (χ^2^ = 6.06, *p* = 0.048) and a higher rate of metastasis (χ^2^ = 10.38, *p* = 0.001). The results showed that KAP1 expression correlated with aggressive clinical features in ovarian cancer. Additionally, positive correlation was identified between KAP1 expression levels and age of patients (χ^2^ = 4.13, *p* = 0.042).

**Table 2:** Correlation between KAP1 expression and clinicopathological features in 111 patients with epithelial ovarian cancer.

Variables	*n*	KAP1 Expression		*χ*^2^	*p*
		Low	High		
Age (years)					
<55	56	29	27	4.13	0.042
≥55	55	18	37		
Clinical stage					
Early (stage I–II)	39	26	13	14.57	0.000
Advanced (stage III–IV)	72	21	51		
Grade					
I	18	11	7	6.06	0.048
II	22	12	10		
III	71	24	64		
Ascites					
No	37	20	17	3.12	0.077
Yes	74	27	47		
Metastases					
Negative	22	16	6	10.38	0.001
Positive	89	31	58		
Histology type					
Serous	59	23	36	5.35	0.148
Endometrioid	37	14	23		
Mucinous	9	7	2		
Clear cell	6	3	2		

**Figure 3:**
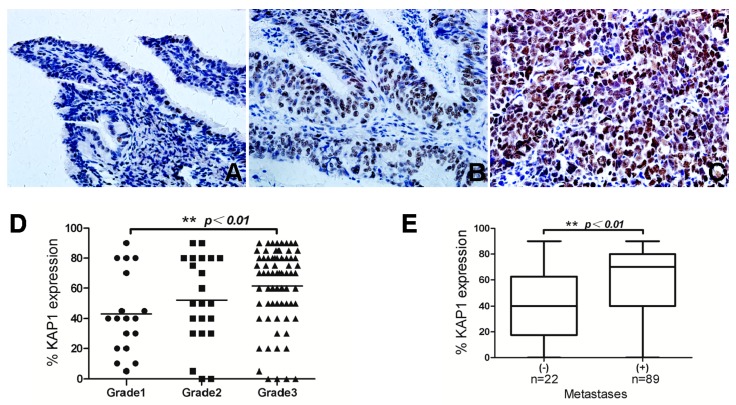
KAP1 expression levels in different pathological grade (**A**) grade 1; (**B**) grade 2; (**C**) grade 3; (**D**) Significant differences in KAP1 expression levels between grade 1 and grade 3 and (**E**) KAP1 expression levels in groups of different metastasis status. (** *p* < 0.01).

### 2.4. Survival Analysis

**Figure 4:**
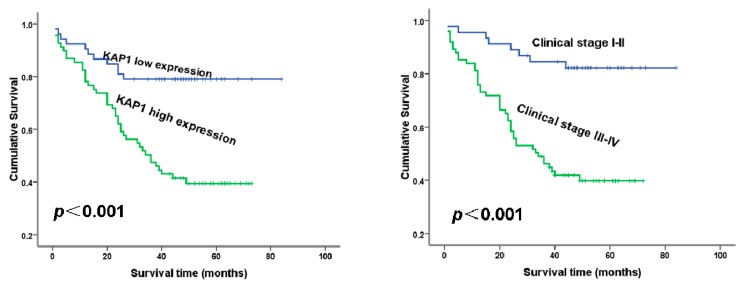
The prognostic effects of KAP1 expression levels in ovarian cancer patients. (**A**) Patients with high KAP 1 expression showed significantly shorter overall survival than those with low KAP 1 expression (*p* < 0.001) and (**B**) Patients with stage I–II disease showed significantly longer overall survival than those with stage III–IV disease (*p* < 0.001).

Kaplan-Meier survival analysis showed that the survival rate was significantly lower in the KAP 1 high-expression group than in the low-expression group (*p =* 0.00; Figure 4A). There was a statistically significant difference in ovarian cancer-specific survival between tumors with high* vs.* low clinical stage (*p* = 0.00; Figure 4A), which was consistent with the previous study [16].

In the multivariate analysis, KAP1 was a significant predictor of the survival (*p* = 0.031) when entered into a model containing all clinicopathologic variables with significance in Kaplan-Meier survival analysis (Table 3).

**Table 3:** Univariate and multivariate analysis of survival in all population.

Variables	Univariate	Multivariate	
	*p*-Value	HR (95% CI)	*p*-Value
Age, years (<55* vs.* ≥55)	0.949	ND	ND
Metastases	0.001	0.395 (0.115–1.362)	0.142
Stage (I/II* vs.* III/IV)	0.000	0.405 (0.181–0.905)	0.028
Ascites (No* vs.* Yes)	0.739	ND	ND
Grade (Well* vs.* Poor)	0.840	ND	ND
KAP1 (Low* vs.* High)	0.000	0.463 (0.230–0.931)	0.031

Abbreviations: ND, not done.

### 2.5. Loss of KAP1 Inhibits Ovarian Cancer Cell Growth and Migration

**Figure 5:**
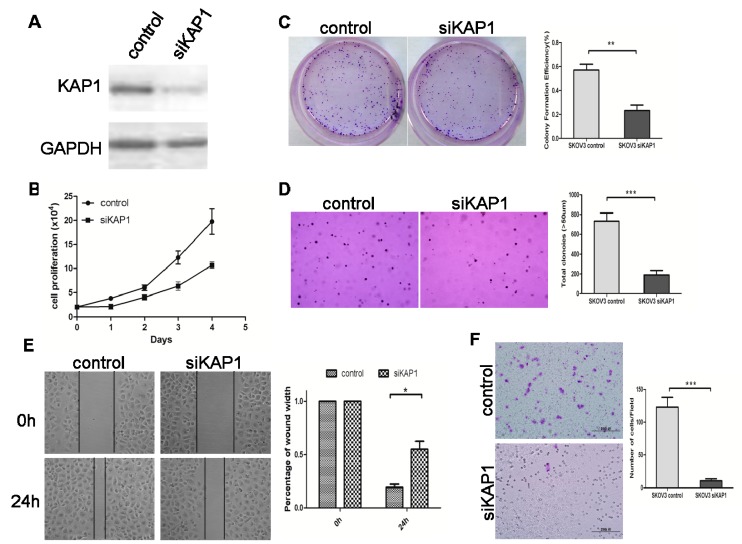
Downregulated expression of KAP1 inhibits cell growth and migration in SKOV3 cells. (**A**) western blot indicates KAP1 interference efficiency; (**B**) MTT assay at 0, 24, 48, 72 and 96 h after transfection; (**C**) representative images of two-dimensional culture of cells; (**D**) representative images of soft agar colony formation assay of cells (100×); (**E**) photographs representing the cells migrated into the wounded area, and histogram showing the relative migration distance of cells in the wound-healing assay (200×) and (**F**) transwell migration assay (100×). (* *p* < 0.05; ** *p* < 0.01; *** *p* < 0.001).

**Figure 6:**
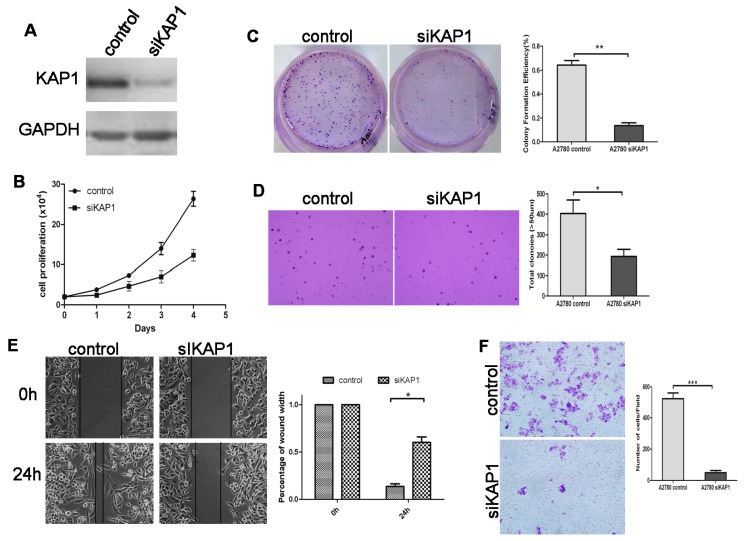
Downregulated expression of KAP1 inhibits cell growth and migration in A2780 cells. (**A**) western blot indicates the KAP1 interference efficiency; (**B**) MTT assay at 0, 24, 48, 72 and 96 h after transfection; (**C**) representative images of two-dimensional culture of cells; (**D**) representative images of soft agar colony formation assay of cells (100×); (**E**) photographs represent cells migrated into the wounded area, and histogram shows the relative migration distance of cells in the wound-healing assay (200×) and (**F**) transwell migration assay (100×). (* *p* < 0.05; ** *p* < 0.01; *** p < 0.001).

Because KAP1 was obviously overexpressed in advanced stage ovarian cancer, we investigated the effect of KAP 1 on ovarian cancer cell growth and anchorage- independent growth. siRNA was used to deplete expression of KAP1 in ovarian cancer cell lines SKOV3 and A2780 (Figure 5A and Figure 6A). The MTT cell proliferation assay demonstrated that lowering KAP1 expression significantly inhibited ovarian cancer cell growth by 50% in the SKOV3 siKAP1 group (*p* < 0.01), and 60% in the A2780 siKAP1 group (*p* < 0.001) when compared with the control group (Figure 5B and Figure 6B). The stable KAP1 knockdown in SKOV3 cell line exhibited a two to three-fold decrease in cell growth based on the focus formation assay (*p* < 0.01) (Figure 5C) and a three to four-fold decrease in colony formation using the anchorage-independent growth assay (*p* < 0.01) (Figure 5D), and similar results were found in the A2780 cell line, (Figure 6C,D). In summary, these findings suggest that KAP1 can promote both the anchorage-dependent and -independent growth of ovarian cancer cells.

We also studied the functional effect of KAP1 in ovarian cancer cell migration. In a wound-healing assay, the relative migration distances of SKOV3-siKAP1 cells and A2780-siKAP1 cells were significantly shorter than control cells (Figure 5E and Figure 6E). In addition, we studied the effect of KAP1 on ovarian cancer cell migration using a transwell migration assay. Stable depletion of endogenous KAP1 in SKOV3 and A2780 cells using siRNA inhibited the cell migration (all *p* < 0.001) (Figure 5F and Figure 6F). These results indicate that downregulation of KAP1 may inhibit the aggressiveness of ovarian cancer and explain why its level is progressively increased in advanced stage and high-grade ovarian cancers.

## 3. Discussion

To our knowledge, this is the first report to quantify KAP1 expression levels by immunohistological staining and investigate their clinical relevance in ovarian cancer. In the present study, we found that the expression levels of KAP1 in ovarian cancer tissues and borderline tumor were higher than normal ovarian tissues. High levels of KAP1 expression were associated with aggressive clinical conditions in ovarian cancer. Also, higher expression of KAP1 was an independent prognosis factor for ovarian cancer patients. Loss of KAP1 inhibited ovarian cancer cell growth and migration.

Ovarian cancer is located deeply within the pelvis and lacks typical early symptoms; many patients are diagnosed at an advanced stage when the tumor has metastasized into the peritoneal cavity or to distant locations. Although surgical treatment and chemotherapy of ovarian cancer have been improved in recent years, the prognosis of ovarian cancer remains poor. The five-year survival in patients with advanced ovarian cancer is less than 30%. Current diagnostic methods for ovarian cancer mainly include pelvic examination, transvaginal ultrasound, and serum CA125. However, these methods are not sensitive or sufficiently specific to diagnose ovarian cancer at an early stage. Consequently, finding a diagnostic marker with high sensitivity and specificity for early detection remains a major clinical challenge.

KAP1 is an essential partner in several multiple-protein complexes and involves a wide range of biological processes. There are several papers about KAP1 expression in cancer tissues and blood serum, but not in ovarian cancer. The level of KAP1 mRNA expression was found to be an independent diagnostic marker of malignant thyroid neoplasms [17]. Upregulation of the KAP1 gene in cancer tissues has been shown in gastric cancer and is associated with poor prognosis [18], and its upregulation was also detected in peripheral blood of gastric cancer patients [19]. Previous studies showed that KAP1 was overexpressed in both liver and peritoneal metastases from patients with colorectal adenocarcinoma, melanoma and malignant thyroid neoplasms [20]. KAP1 also showed significant increase in colorectal carcinomatous epithelium compared with the adenomatous epithelium [21]. The level of serum KAP1 in patients with melanoma has previously been shown to be significantly higher than in controls, leading to the speculation that serum levels of KAP1 may be a new useful marker for melanoma progression [21]. The expression of the KAP1 gene was significantly higher in cancerous tissues than in noncancerous tissues and was a new marker to predict metastasis and prognosis in early stage non-small cell lung cancer patients [22]. Chen *et al.* showed that KAP1 gene expression was downregulated in the early stages of lung cancer as a tumor suppressor, regulating HDAC1/E2F interactions [23], which suggests a complex role for KAP1 in lung cancer; KAP1 may have both tumor-promoting and tumor-suppressing functions.

However, there have been a number of indirect indications in recent studies that suggest KAP1’s role to promote tumorigenesis in addition to cancer cell migration. KAP1 knockdown resulted in an impaired proliferation rate and increased G1-phase cell cycle fraction [22]. Furthermore, KAP1 was observed to inhibit P53 acetylation and to promote P53 ubiquitination by interacting with MDM2 [24], suggesting KAP1 also plays a role in cancer by inhibiting p53. KAP1 also suppressed the cyclin-dependent kinase inhibitor P21 [25,26]. The results of the present study are consistent with the disruption of the G1/S checkpoint via inhibition of P53 and P21 function. Furthermore, KAP1 may play a role in inducing EMT [15], which is considered to be involved in migration, tumor invasion, and dissemination. These results indicate that KAP1 is a universal corepressor.

Lin *et al.* showed the first direct evidence to demonstrate that KAP1 has the ability to promote tumor metastasis both* in vitro* and* in vivo* [25], which is in accordance with our results. In our study, the expression level of KAP1 was higher in ovarian cancer samples than non-tumor ovarian tissues (Figure 1A–D), and the positive rates of KAP1 expression were significantly higher in ovarian epithelial cancer (55.7%) and borderline tumor (20.0%) than in normal ovarian tissue (5.0%) (all *p* < 0.01), which suggested that KAP1 may play an important role in ovarian cancer genesis. Furthermore, the expression level of KAP1 protein was significantly associated with a higher clinical stage (χ^2^ = 14.57, *p* < 0.0001), higher histological grade (χ^2^ = 6.06, *p* = 0.048) and a higher rate of metastasis (χ^2^ = 10.38, *p* = 0.001). These results showed that KAP1 expression is correlated with aggressive clinical features and promoted the development of ovarian cancer. We obtained similar results in cell lines SKOV3 and A2780 through inhibition of KAP1 expression by siRNA, and loss of KAP1-inhibited ovarian cancer cell growth and migration.

## 4. Materials and Methods

### 4.1. Patients and Tissue Samples

One hundred and eleven ovarian epithelial cancers, 15 ovarian borderline tumors, and 20 normal ovarian tissues were obtained from the Department of Pathology, Tianjin Medical University Cancer Institute and Hospital in 2006. All tissue sections were examined by specialists to make a final diagnosis. Histopathological diagnoses were made using the World Health Organization criteria. The classification of cancer stage and grade was according to the International Federation of Gynecology and Obstetrics (FIGO, 2009). Clinicopathological data were collected including age, histology type, ascites, metastases status and tumor grade. All patients’ characteristics were summarized in Table 1.

The age range (median) was 27–83 years (54 years) in the ovarian tumor group; 18–77 years (45 years) in the borderline ovarian tumor group; and 23–81 years (43 years) in the normal group. There were no statistically significant differences in the ages of these groups (*p* > 0.05).

### 4.2. Antibodies

The primary antibodies used in this study were listed as follows: rabbit anti-KAP1 (Santa Cruz Biotechnology, Santa Cruz, CA, USA), mouse anti-β-actin (Santa Cruz Biotechnology), mouse anti-GAPDH (Santa Cruz Biotechnology). The anti-mouse secondary antibodies (Santa Cruz Biotechnology), and anti-rabbit secondary antibodies (Zhongshan Goldbridge Biotechnology, Beijing, China) were purchased for western blot.

### 4.3. Immunohistochemistry Staining and Evaluation

Sections 4 μm in thickness were deparaffinized and rehydrated with xylene and graded alcohol solutions. After PBS wash, endogenous peroxidase activity was quenched by 3% hydrogen peroxide, and sections were boiled in 10 mM citrate buffer (pH 6.0) for 3 min in an autoclave sterilizer followed by cooling at room temperature for more than 20 min. After PBS rinsed, sections were incubated with primary antibodies (1:75 dilution in antibody diluent, Zhongshan Goldbridge Biotechnology CO., Ltd., Beijing, China) for 18 h at 4 °C. After PBS rinse, sections were incubated with PV6001 or PV6002 (Zhongshan Goldbridge Biotechnology CO., Ltd., Beijing, China) for 30 min at 37 °C and stained with DAB for 1 to 2 min. The slides were counterstained with hematoxylin, dehydrated with ethanol, cleared with xylene, and mounted in neutral gum. Control sections were incubated with PBS instead of a primary antibody.

We chose five high-power fields in serial sections from each slice, scored them, and estimated the mean percentage of chromatic cells. Patients with KAP1 expression levels of ≤50% in tumor tissues were assigned to the low-expression group (*n* = 47), whereas those with values >50% were assigned to the high-expression group (*n* = 64). The cutoff between the two groups was defined by the mean value of KAP1 expression in cancerous tissue.

### 4.4. Cell Culture

Cell lines SKOV3 and A2780 were obtained from ATCC (Rockville, MD, USA), and the cell line was cultured in minimum essential medium (MEM) supplemented with 10% FBS (Gibco, Invitrogen Life Technologies, Carlsbad, CA, USA) inside an incubator containing 5% CO_2_ at 37 °C.

### 4.5. RNA Interference Assay

The KAP1 siRNA sequence 5'-GCGATCTGGTTATGTGCAATT-3', and the control siRNA sequence was synthesized by Shanghai Genechem Co., Ltd. (Shanghai, China). The siRNAs were synthesized and subcloned into a lentiviral siRNA vector Plko.1-Amp/puromycin. The transfected cells were cultured for 48 h, and expression protein level were confirmed with Western-blot analysis in cells. The day before transfection, 1 × 10^5^ cells were placed in 35 mm dishes in DMEM supplemented with 10% fetal bovine serum and without antibiotics.

### 4.6. Western Blot

All agents were purchased from Santa Cruz Biotechnology. Total protein was obtained using a lysis buffer (1% SDS, 10 mM Tris-Hcl, pH 7.6, 20 μg/mL aprotinin, 20 μg/mL leupeptin and 1 mM AEBSF) and the protein concentration was measured with Bradford method. Twenty micrograms of protein were separated on a 10% SDS-PAGE gel and blotted onto a PVDF membrane. After blocking with 5% fat-free milk in TBS-Tween 1 h, the membrane was incubated with antibody for 1 h at room temperature. After washing thrice with TBS-Tween, the membrane was labeled with horseradish peroxidase-conjugated secondary antibody for 1 h at room temperature. Blots were developed with a DAB kit. β-actin (sc-1616) was used as an internal control. The bands for samples were analyzed with a gel imaging system (Kodak, Rochester, NY, USA). The gray-scale ratio of KAP1 to β-actin in every sample was considered as the relative protein level.

### 4.7. MTT Assay for Cell Proliferation

Seventy-two hours after transfection, siPAK1 and control cells were seeded in sextuplicate in 96 well plates, at a density of 3000 cells/well and incubated for 0, 24, 48, 72 and 96 h. At the end of incubation, 20 µL of 5 mg/mL MTT (3-(4,5-dimethylthiazol-2-yl)-2,5-diphenyl-2H-tetrazolium bromide, Sigma, St. Louis, MO, USA) were added to each well. The plates were incubated in a humidified incubator at 37 °C, under 5% CO_2_ for 4 h, following which 150 µL dimethyl sulfoxide was added. The plates were gently agitated until the formazan was completely dissolved, and the absorbance was measured at 490 nm wavelength.

### 4.8. Clonogenic Survival Assay

Viable cells (500/well) were seeded on 6-well plates and incubated for 7–14 days. After this they were then fixed with methanol and stained with gentian violet. Colonies containing more than 50 cells were scored as surviving cells. Each surviving fractions were corrected using these cell survivals. The survival curves were fitted to a linear-quadratic model.

### 4.9. Soft Agar Colony Formation Assay

Soft agar assays were constructed in 6-well plates. 1 × 10^4^ cells were plated in 0.4% agarose on top of a 1% agarose base supplemented with complete medium. A further 1 mL of 1× media without agarose was added on top of the growth layer on day 0 and again on day 14 of growth. Cells were allowed to grow at 37 °C for 4 weeks and total colonies were counted. The pictures were taken by digital camera or microscope and the number of colonies were counted by Quantity One software.

### 4.10. Wound-Healing Assay

Cells were seeded in 6-well plates and grown overnight in culture medium containing 10% FBS to reach 90% confluence. Cell monolayer was wounded by scratching with a 20 μL pipette tip, followed by washing three times with serum-free medium. Then cells were incubated in serum-free culture medium. For each well, images of the scratch were taken at 0 and 24 h using an inverted microscope at 10× magnification. The distances of cell migration were calculated by subtracting the distance between the lesion edges at 24 h from the distance measured at 0 h. The relative migrating distance of cells is measured by the distance of cell migration/the distance measured at 0 h.

### 4.11. Cell Migration Assay

The cell migration analysis was carried out using transwell inserts (8.0 mm pore size, Costar, Cambridge, MA, USA). The cells were seeded at a density of 50,000 per well and then in 200 μL of serum-free medium for the stimulation. The medium containing DMEM supplemented with 0.1% fetal bovine serum was placed in the lower chamber in the presence of epidermal growth factor (EGF) (20 ng/mL, R&D Systems, Minneapolis, MN, USA). After incubation for 6 h, noninvading cells on the top of each Transwell were scraped off with a cotton swab. Cells that had migrated to the other side were fixed with 2.5% glutaraldehyde (Wako, Tokyo, Japan) and stained with crystal violet (Wako). The number of migrated cells was manually counted with a light microscope (KX4, Olympus, Tokyo, Japan). The sum of the numbers of cells in five areas was used as the migrated cell number, and expressed as a percentage of the control value. These experiments were repeated at least three times, and significant differences among treatments were assessed by ANOVA followed by Tukey’s test.

### 4.12. Statistical Methods

SPSS 16.0 was used to evaluate the data. The χ^2^ test was used to assess the difference in KAP1 expression and the pathological and clinical factors between normal ovarian tissues and ovarian cancer tissues. Survival was analyzed using the Kaplan-Meier analysis. Cox’s proportional hazard regression model was used for multivariate survival analysis of prognostic factors. The standard two-tailed independent samples *t*-test was performed to compare the differences of the KAP1 protein in two groups. The significance level was defined as* p* < 0.05.

## 5. Conclusions

In conclusion, we have demonstrated the biological and clinical significance of KAP1 expression in ovarian cancer. Overexpression of KAP1 was likely to be an essential contributor to growth promotion of ovarian cancer. Detection of KAP1 protein expression levels was demonstrated to be a sensitive tool for predicting the metastasis and prognosis in early-stage patients.
